# Supplementary material for: Uncovering hidden cancer self-dependencies through analysis of shRNA-level dependency scores
Source: Sci Rep. 2024 Jan 9;14:856. doi: 10.1038/s41598-024-51453-5 (PMC10776685; doi:10.1038/s41598-024-51453-5)
Supplement: Supplementary file 1 — Supplementary Information. [file 41598_2024_51453_MOESM1_ESM.pdf]

Supplementary figures and tables

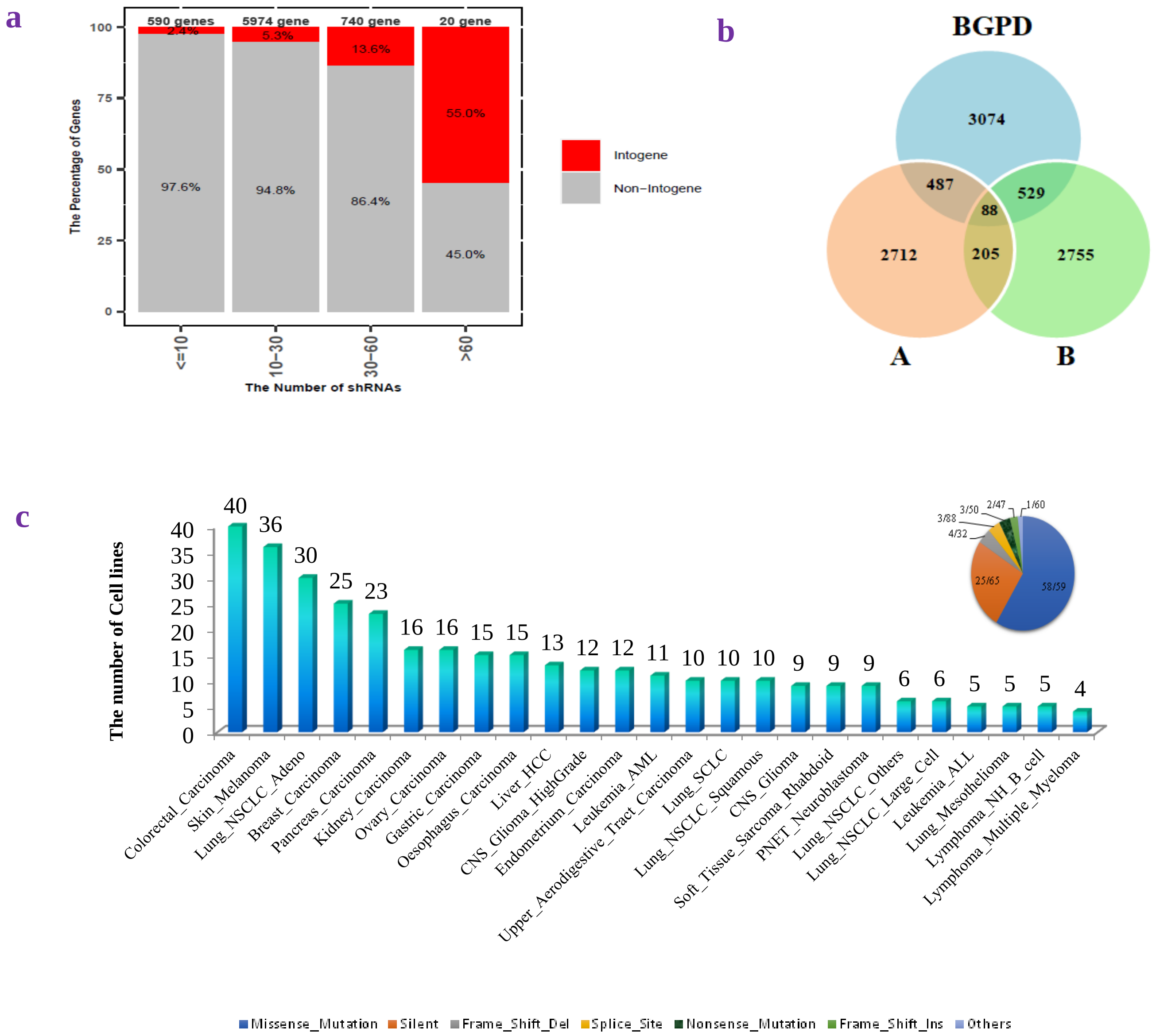

Supplementary Figure S1. Exploring cell lines, shRNAs, and variant types. **a**. The number of shRNA designed for two groups of genes: IntOGen and Non-IntOGen. **b**. Venn diagram indicating the number of genes in three different pools namely A, B, and BGPD in Project DRIVE. c. The number of cell lines across cancer types in Project DRIVE. The pie chart shows the percentage of each variant type of genes in cell lines, including missense, silent, frameshift deletion, splice site, nonsense, frameshift insertion, and others.

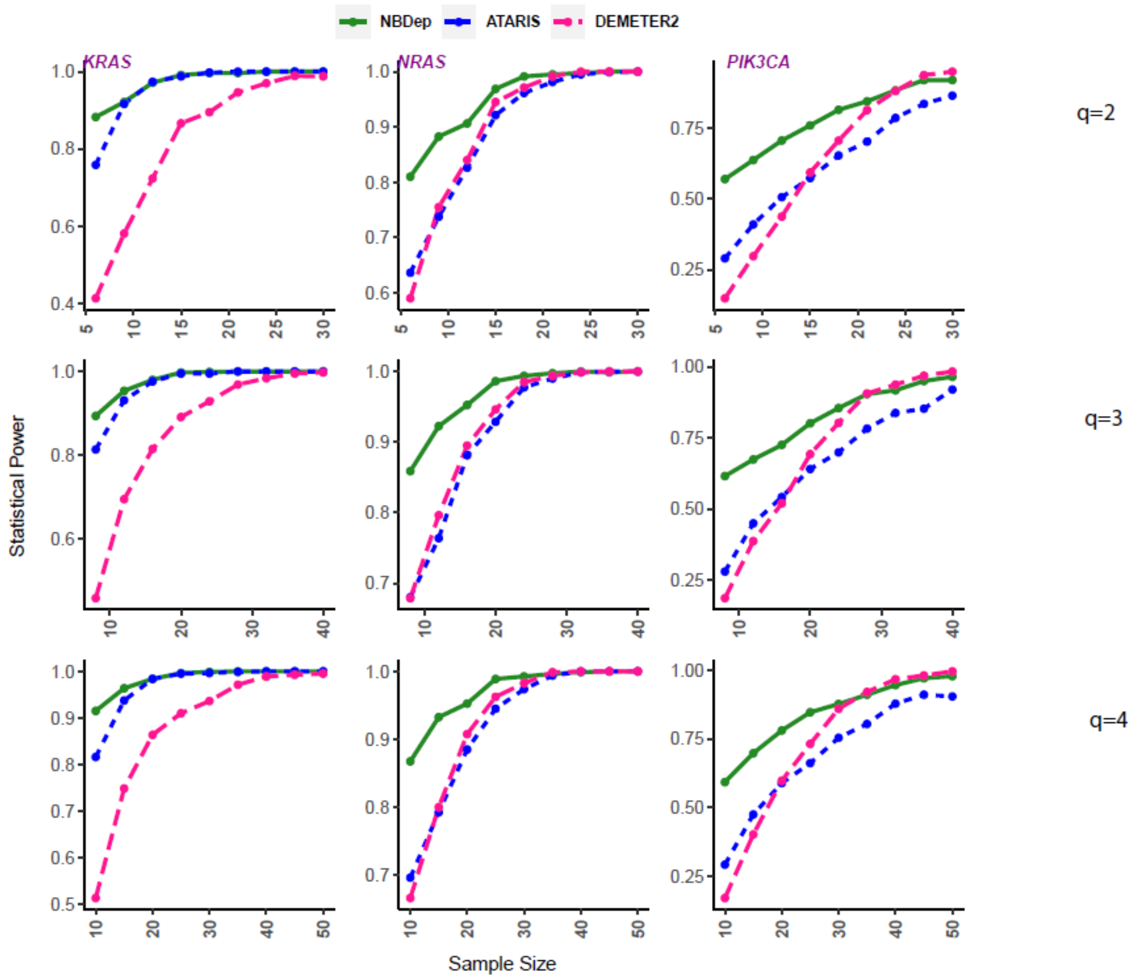

Supplementary Figure S2. Comparison of statistical power between APSiC method applied to ATARiS and DEMETER2 scores and the NBDep algorithm for *KRAS*, *NRAS*, *PIK3CA* across various sample sizes with q=2-4.

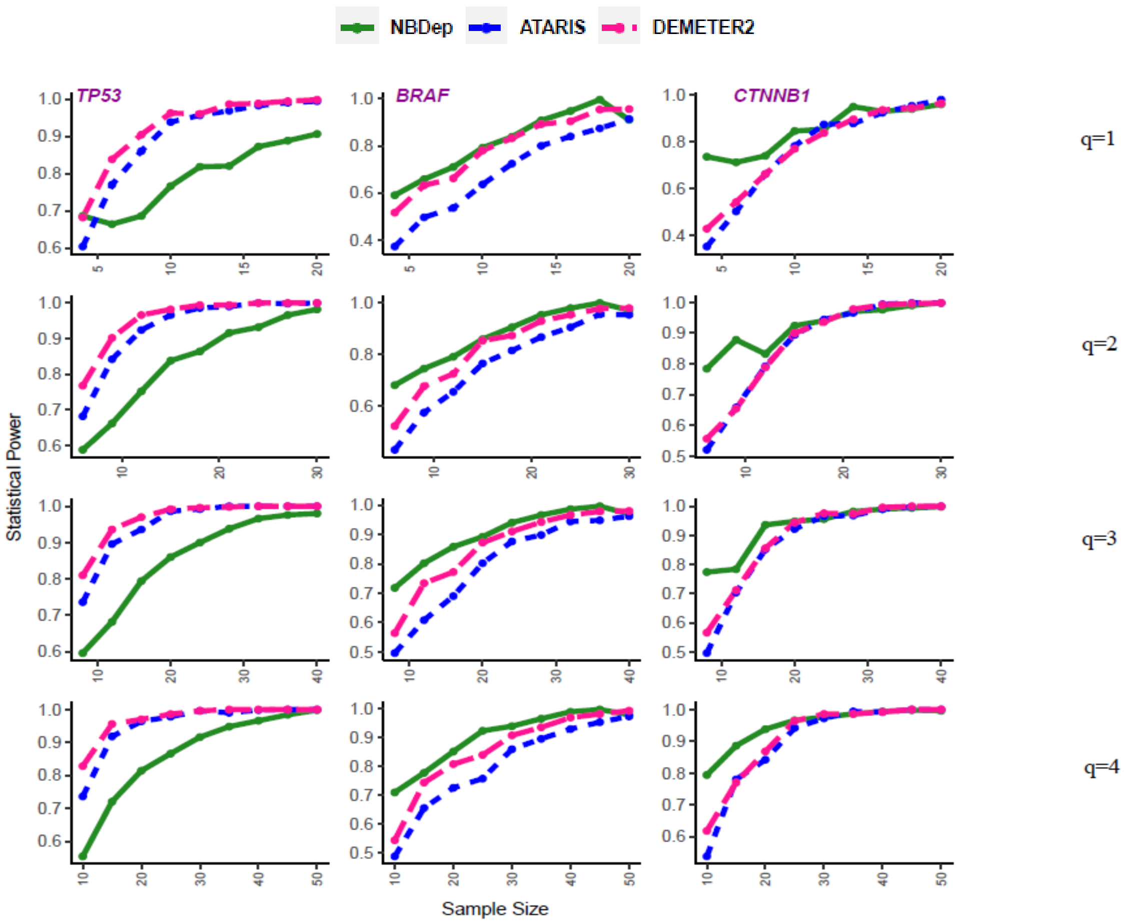

Supplementary Figure S3. Comparison of statistical power between APSiC method applied to ATARiS and DEMETER2 scores and the NBDep algorithm for *TOP2A*, *HRAS*, *TP53* across various sample sizes with q=1-4.

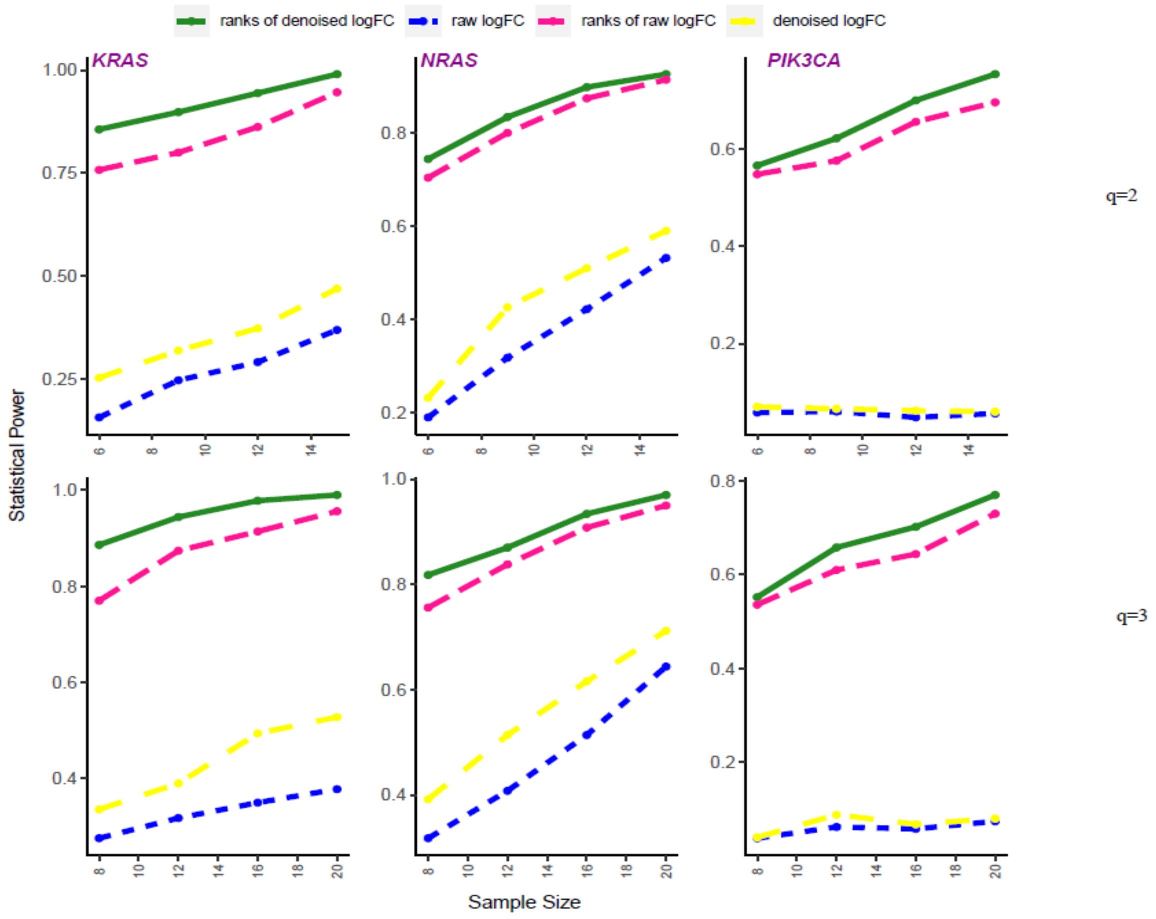

Supplementary Figure S4. Comparison of statistical power across four models namely NBDep, linear mixed-effect model on raw logFC, linear mixed-effect model on denoised logFC, and negative binomial mixed-effect model on ranks of raw logFC for *KRAS*, *NRAS*, and *PIK3CA* across various sample sizes with q=2,3.

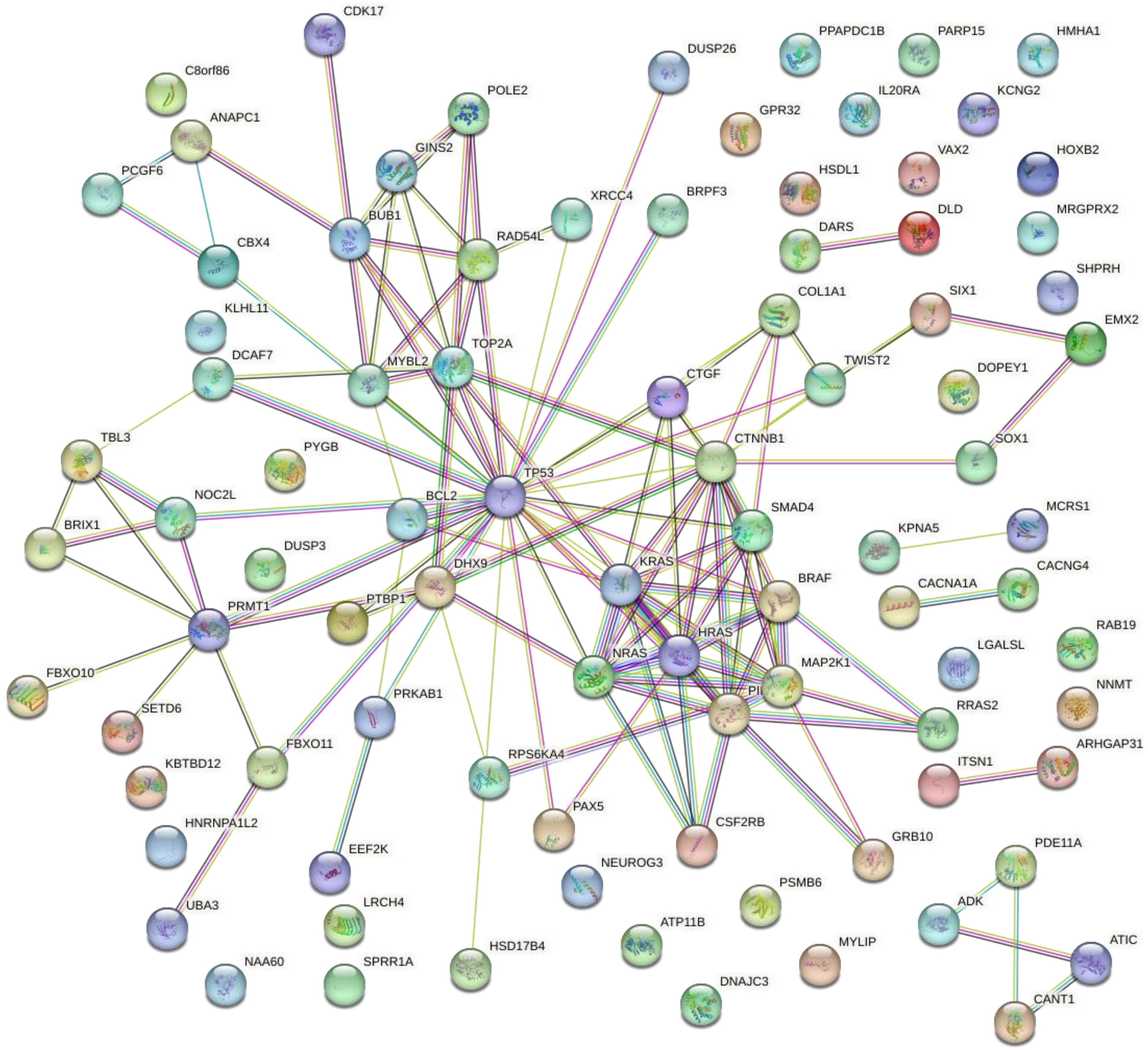

Supplementary Figure S5. The Protein-Protein network (PPI) of identified missense genes by NBDep in pan-cancer, extracted from STRING. Top identified genes by NBDep including *KRAS*, *NRAS*, *PIK3CA*, *BRAF*, *HRAS*, *TP53*, *TOP2A*, and

CTNNB1 are densely connected in the PPI network. *ANAPC1*, a novel gene identified by NBDep, has coexpressed with *BUB1* which is coexpressed with *TP53* and *TOP2A*, two known cancer driver genes.

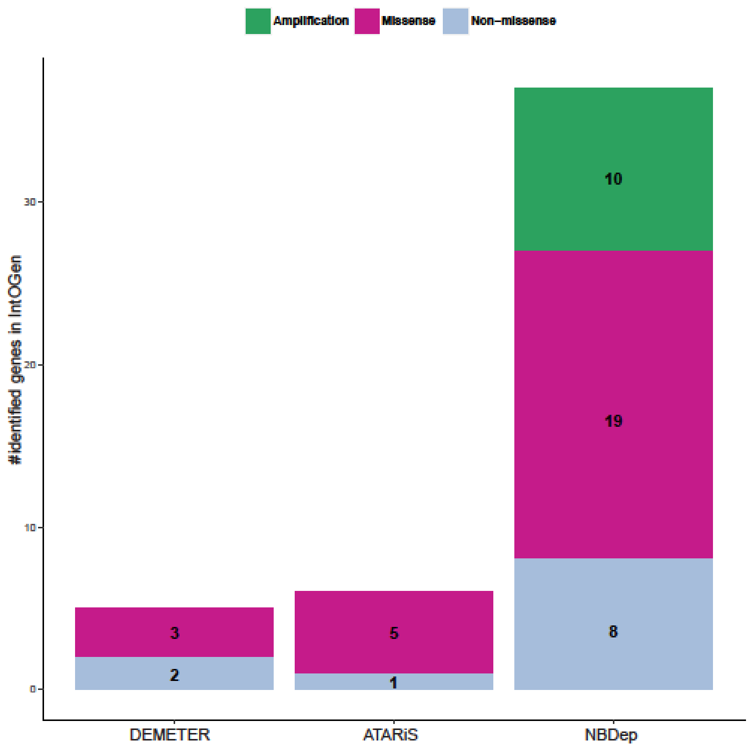

Supplementary Figure S6. Number of genes identified by three methods (applying APSiC on ATARiS and DEMETER scores as well as NBDep) in three alterations (amplification, missense, and non-missense) that are common with IntOGen in any cancer-specific analysis.

Supplementary Table 1: Identified genes by the NBDep algorithm in three alterations (amplification, missense, and non-missense) in both pan-cancer and 26 cancer types.
